# Nonpapillary Prone Endoscopic Combined Intrarenal Surgery (ECIRS): Five-Year Experience and Outcomes from a High-Volume Center

**DOI:** 10.3390/jcm13020621

**Published:** 2024-01-22

**Authors:** Panagiotis Kallidonis, Theodoros Spinos, Vasileios Tatanis, Anna Skarimpa, Theofanis Vrettos, Paraskevi Katsakiori, Evangelos Liatsikos

**Affiliations:** 1 Department of Urology, University of Patras Hospital, 26504 Patras, Greece; thspinos@otenet.gr (T.S.); tatanisbas@gmail.com (V.T.); annitaskar@gmail.com (A.S.); vkatsak@upatras.gr (P.K.); liatsikos@yahoo.com (E.L.); 2 Department of Anesthesiology and ICU, University of Patras, 26504 Patras, Greece; teovret@gmail.com; 3 Department of Urology, Medical University of Vienna, 1090 Vienna, Austria

**Keywords:** stone disease, endourology, ECIRS, prone position, noncalyceal puncture

## Abstract

Endoscopic combined intrarenal surgery (ECIRS) provides simultaneous retrograde and percutaneous access to the upper urinary tract. The purpose of this study is to present revised data, tips and tricks, and technique modifications arising from our five-year experience with ECIRS. The data of 62 patients who underwent nonpapillary prone ECIRS from January 2019 to November 2023 were prospectively collected. All cases were performed in the prone position. Inclusion criteria were complex stone cases with stones in multiple calyces requiring either multiple accesses or multiple sessions to achieve stone-free status. Patients’ mean age was 54.4 ± 12.39 years, while the mean stone size was 39.03 ± 13.93 mm. The mean operative time was 51.23 ± 17.75 min. Primary and final stone-free rates were 83.8% and 90.3%, respectively. In total, nine patients presented with postoperative complications, which were all Grade II ones. The holmium-YAG laser type during retrograde lithotripsy was associated with significantly shorter operative times compared to the thulium fiber laser. Nonpapillary prone ECIRS is a feasible, safe, and efficient approach for patients with specific stone and anatomy characteristics. The implementation of more, higher-evidence studies is of utmost importance so that safer conclusions can be drawn.

## 1. Introduction

Urolithiasis refers to the presence of stones in the urinary tract and constitutes a common disease worldwide as its incidence reaches 7–13% in North America, 5–9% in Europe, and 1–5% in Asia [1,2,3]. Several factors, including age, gender, occupation, genetics, diet, fluid intake, geography, and climate, can affect the regional incidence of stone disease [4]. Interestingly, the incidence of urolithiasis is dramatically increased in patients in the age range of 20–50 years, thus representing over 70% of the population diagnosed with urolithiasis [5]. The treatment of urolithiasis may involve both surgical and non-surgical interventions [6]. The goal of surgical therapy is to achieve stone-free status (SFS). Recent developments in surgical equipment technology, such as the minimization of instruments and the elevated power of energy sources, have led to the reduced use of open surgical approaches and thus diminished postoperative pain and complication rates [7]. Shockwave lithotripsy (SWL), ureteroscopy (URS), and percutaneous nephrolithotomy (PCNL) represent the main surgical treatment approaches. All of them constitute excellent treatment options with different efficacy, indications, and complication rates [6,7,8].

In 2008, Scoffone et al. introduced a new combined endoscopic method, endoscopic combined intrarenal surgery (ECIRS). ECIRS provides the capability of simultaneous transurethral and percutaneous access to the upper urinary tract, namely the contribution of retrograde intrarenal surgery (RIRS) during PCNL [6]. In the original technique, the patient was positioned in the Galdakao-modified supine Valdivia (GMSV) position, which provides transurethral access to the urinary system [9]. The authors declared that it was the adoption of this position and the abandonment of the prone position that led them to routinely combine PCNL with retrograde URS [10]. The main purpose of ECIRS is to achieve maximal stone-free rates (SFRs) with a single procedure. As flexible ureteroscopy (URS) enables access to stones in calyces, which cannot be reached with a nephroscope, ECIRS can be useful in cases with anatomical abnormalities (e.g., renal rotation, horseshoe kidneys, and duplicated collecting system), difficulties in the angle of the pelvicalyceal system, or staghorn stones [11]. Several studies have demonstrated the feasibility, safety, and efficacy of ECIRS [10,12,13]. A recently performed systematic review reported that ECIRS is associated with an SFR and a complication rate ranging from 61 to 97% and from 5.8 to 44%, respectively [14]. Most of the complications were stratified as grade I or II according to the Clavien–Dindo classification [15]. In the same study, when ECIRS was compared to PCNL alone, it was found to have comparable operative times, higher SFRs, reduced complication rates and fluoroscopy time, less need for multiple punctures, and shorter hospitalization [14]. In a previous publication, we reported the safety and efficacy of nonpapillary prone ECIRS and presented the outcomes of all ECIRS procedures performed in our center from 2019 to 2021 [16]. The purpose of this study is to present revised data, tips, and tricks, as well as technique modifications, arising from our five-year experience with ECIRS (2019–2023).

## 2. Materials and Methods

### 2.1. Study Design and Study Population

This study represents a retrospective analysis of prospectively collected data. Data of 62 consecutive patients who underwent nonpapillary prone ECIRS in the Department of Urology, University Hospital of Patras, Greece, from January 2019 to November 2023 were prospectively collected. Inclusion criteria were complex cases with stones in multiple calyces requiring either multiple accesses or multiple sessions to achieve SFS. All procedures were performed by two experienced and high-volume stone (performing > 500 endoscopic procedures per year) surgeons (E.L. and P.K.) with expertise in both PCNL and RIRS.

### 2.2. Preoperative Examinations and Diagnostic Work-Up

Patients’ baseline characteristics such as age, gender, body mass index (BMI), presence of anatomical variation (malrotation, horseshoe kidney, or duplicated systems), and American Society of Anesthesiologists (ASA) score [17] were prospectively collected. Preoperative work-up of the patients included history, clinical examination, and routine laboratory tests (hemoglobin count, creatinine level, and chest X-ray). Preoperatively, all patients were assessed by a cardiologist and a pneumonologist to determine the risk of intraoperative and postoperative complications. Prior to the surgery, stone characteristics were evaluated with the use of kidneys, ureters, and urinary bladder (KUB) X-ray, KUB ultrasound (US), and KUB computerized tomography (CT). Stone size was estimated by the maximal stone diameter. Partial staghorn calculi were considered as the stones that filled the renal pelvis and at least two of the calyces, whereas complete staghorn calculi were considered as the stones that filled the renal pelvis and the upper, the middle, and the lower calyces.

### 2.3. Surgical Technique and Intraoperative Characteristics

**Figure 1:**
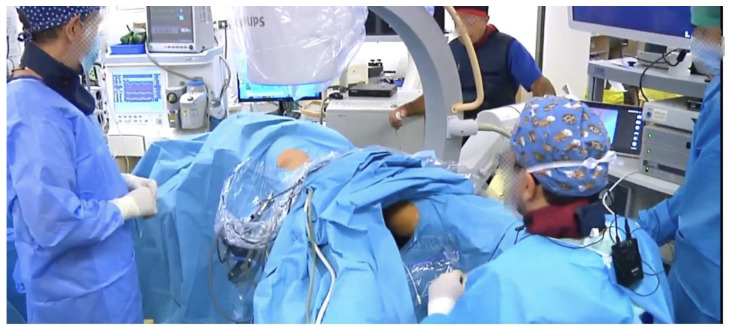
The setup of the operation room. The position of the patient is prone split-leg. The monitors are on the contralateral side next to the c-arm. The flexible RIRS team stands on the lower side of the patient. The PCNL team stands on the ipsilateral side.

All procedures were performed under general anesthesia. The patients were either prestented (underwent ureteral stent placement before surgery) or non-prestented (received alpha blockers one week preoperatively). All patients were placed in lithotomy position either prior to the beginning of the procedure or in some cases the day before the surgery. An open-end ureteral catheter was inserted using a rigid cystoscope. The ureteral catheter was positioned at the level of the ureteropelvic junction, and a retrograde pyelogram was acquired. The patient was then placed in the prone position (Figure 1).

**Figure 2:**
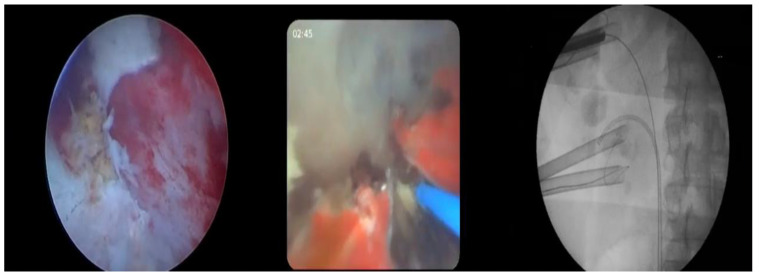
Simultaneous view of the utilized screens. PCNL screen on the left side. Flexible RIRS screen in the middle. The c-arm screen on the right side shows both the instruments involved.

A nonpapillary renal puncture was carried out near the lower or middle calyces, and Amplatz dilators (Amplatz renal dilator set, COOK Medical, Bloomington, IN, USA) were used to create tract dilation. In some cases, a second or a third puncture was deemed necessary to achieve optimal SFRs. Afterward, a 30 Fr (standard PCNL) or a 22 Fr (mini PCNL) access sheath was introduced using a two-step or a one-step technique, respectively (Figure 2). In all procedures performed post-January 2022, a 22 Fr access sheath was used. In cases where a 30 Fr access sheath was inserted, a 26 Fr scope was used, while in cases where a 22 Fr access sheath was inserted, an 18 Fr rigid nephroscope was employed. The technical details of our PCNL technique (puncture and tract dilation times) have previously been described [18]. As far as RIRS is concerned, a dual-lumen ureteral catheter was inserted followed by the introduction of a second guidewire. A ureteral access sheath (UAS) (Flexor, COOK Medical, Bloomington, IN, USA) was then positioned above the working stiff guidewire. In all prestented patients, a 12/14 Fr UAS was inserted, while in non-prestented patients, a 9.5/11.5 Fr UAS was introduced. After passing a flexible ureterorenoscope through the access sheath, pyeloscopy was carried out (Figure 2). The ureterorenoscope used was either a Flex-XC 11278VS^®^ (Karl Storz, Tuttlingen, Germany) with 12/14 Fr UAS or a PU3033A (PUSEN Medical, Shenzhen, China) with 9.5/11.5 Fr UAS.

The Lithoclast Trilogy^®^ (EMS Medical, Nyon, Switzerland) was predominantly used for the fragmentation of kidney stones. When the nephroscope was unable to reach the stones, retrograde lithotripsy was carried out using one of the following devices: a Holmium-YAG laser device, i.e., Cyber Ho 150^®^ (Quanta System, Samarate, Italy) or MOSES™ Pulse™ 120H (Lumenis Ltd., Yokneam, Israel); or a thulium fiber laser (TFL) device, i.e., a Fiber Dust^®^ laser system (Quanta System, Samarate, Italy). Until December 2021, a Holmium-YAG laser was used for all retrograde lithotripsy cases. However, since January 2022, we have used either a TFL or a Holmium-YAG device. The selection of the laser device was based on the availability of the laser devices in our department. Energy settings ranged from 1 to 2 J while the frequency ranged from 30 to 60 Hz. Our “self-popping” technique for high-power lithotripsy has been described in a previous publication [19]. In some cases, fragments were relocated and presented with the aid of nitinol baskets so that they could later be removed through antegrade percutaneous access.

In all patients, retrograde nephroscopy was subsequently performed to potentially identify remaining stones, and a double J stent (6–8 Fr) was placed. In most standard PCNL cases, a malecot tale tube (20–24 Fr) was inserted, while in most mini PCNL cases, a balloon nephrostomy tube (16–18 Fr) was preferentially used. The drainage was typically removed on the second or third postoperative day. The double J stent removal time ranged from 2 to 4 weeks postoperatively. Intraoperative parameters such as total operative time, number of punctures, access size, and site, as well as laser type during URS, were recorded and assessed. Total operative time was defined as the time from the beginning of the puncture to the drainage placement.

### 2.4. Postoperative Characteristics and Follow-Up

The recorded postoperative data included hospital stay duration, hemoglobin drop, SFRs, need for additional treatments, and complication rates. Patients were typically discharged from the hospital one day after the removal of the malecot catheter or nephrostomy tube. A blood test was routinely performed in all cases on the first postoperative day. The hemoglobin drop was calculated by comparing the preoperative and the postoperative value of hemoglobin. Regarding SFRs, all patients underwent KUB X-ray and US one month after the procedure to determine the presence of residual stones. A non-contrast-enhanced CT (NCCT) was performed in the case of symptomatic patients or abnormal findings on the X-ray or US. NCCT was needed in 32.8% of the patients (*n* = 21). Both the need and the type of additional treatments were recorded. Finally, the complications were stratified based on the Clavien–Dindo Classification System [15].

### 2.5. Statistical Analysis

Data are presented as numbers and percentages. Quantitative variables are reported as mean (standard deviation) or median (interquartile range, ΙQR), while both absolute and relative frequencies are used to report categorical variables. Student’s *t*-test and Pearson’s chi-squared test were employed to compare mean values and proportions, respectively. SPSS v. 21.0 (SPSS Inc., IBM^®^, Chicago, IL, USA, 2012) was used for performing descriptive statistical analyses.

## 3. Results

In total, 62 patients underwent prone nonpapillary ECIRS in our department from January 2019 to November 2023. Multiple variables (age, gender, ASA score, BMI, stone characteristics, and abnormal urinary tract anatomy) and patients’ intraoperative and postoperative data are summarized in Table 1. The patients’ mean age was 54.4 ± 12.39 years, while the mean BMI was calculated to be 26.83 ± 3.51 kg/m^2^. In total, 4.8%, 24.2%, 24.2%, and 46.8% of patients had an ASA score of 0, 1, 2, and 3, respectively. The mean stone size was 39.03 ± 13.93 mm, while 24.2% of the patients presented partial or complete staghorn calculi. A total of thirteen patients presented with renal abnormalities: five patients (8.1%) with horseshoe kidney, three patients with kidney malrotation (4.8%), and five patients (8.1%) with duplicated pelvicalyceal systems. The mean operative time was 51.23 ± 17.75 min. Although most ECIRS procedures were performed with only one puncture, two and three accesses were required in 16.1% and 6.5% of included patients, respectively. A 22 Fr PCNL tract size (mini PCNL) was used in 64.5% of the cases, while a 30 Fr access size (standard PCNL) was used in 35.5% of the patients. The access site was around the anatomical area of the middle and lower calyces in 21% and 79% of the patients, respectively. Regarding the laser type, 79% of the cases were performed with a Holmium-YAG laser while the remaining 21% were performed with a TFL. The median hospital stay was 3 (IQR 2–4) days. The mean hemoglobin loss was 1.23 ± 0.54 g/dL. The primary and final SFRs were 83.8% and 90.3%, respectively. Five patients (8.1%) required an additional treatment, i.e., a second PCNL procedure. Postoperatively, Grade II complications were recorded in nine patients (14.5%). Five patients had transient postoperative fever while four had bleeding that resolved conservatively.

The association between the laser type used and the outcomes is presented in Table 2. A Holmium-YAG laser and TFL were used in 49 and 13 cases, respectively. The mean operative time was significantly shorter with Holmium-YAG than with TFL (48.33 ± 16.11 min vs. 62.15 ± 19.99 min, *p* = 0.011). Early postoperative complications (occurring before hospital discharge), hemoglobin drop, SFRs, and complication rates were comparable between the two groups. As presented in Table 2, the mean stone size did not differ significantly between the two groups.

A comparison of outcomes between the mini PCNL group (40 patients) and the standard PCNL group (22 patients) is provided in Table 3. The mean total operative time was similar between the standard and mini PCNL groups (46.55 ± 16.96 min vs. 53.80 ± 17.85 min, *p* = 0.125). Mean hemoglobin drop, SFRs, and complication rates were also comparable between the two groups. As presented in Table 2, mean stone sizes did not differ significantly between the two groups.

## 4. Discussion

### 4.1. Patient Positioning

Optimal patient positioning during PCNL and ECIRS remains a debatable issue. Prone position has been the standard patient positioning since the first description of the PCNL technique [20], and it is still preferred by 77% of endourologists [21]. Valdivia et al. were the first to present and describe the outcomes of the supine patient position during PCNL [22]. Since then, several modifications have been documented, with each one of them presenting its own benefits and disadvantages [23]. The benefits of the prone position have also been presented in a previous publication by our group. The ability to treat all renal calculi regardless of their size and their position in the pelvicalyceal system, including cases with a large stone burden, and the possibility to treat patients with anatomical abnormalities, such as horseshoe and rotated kidneys, stand out among them. Moreover, a broader surface area enables punctures at almost every site and a wider working area for maximal instrument intrarenal manipulations, and it facilitates intrarenal access in obese patients [24]. As already mentioned, the GMSV position was used during the first report of ECIRS [10]. However, the prone split-leg position has later been described in several cases of ECIRS [25,26,27,28]. A recent study reported comparable outcomes between these two positions [29]. In our department, all PCNL and ECIRS cases are performed in the prone position. Nevertheless, in female patients undergoing ECIRS, a recent modification of our technique, i.e., the standard prone patient positioning and not the split-leg one, has been followed since January 2022. We preplaced the ureteral catheter, and the patient was then placed in prone position without a slit-leg. The procedure was then performed as described in the Materials and Methods Section of our study. No difficulties in gaining and preserving retrograde access have been reported in these cases.

### 4.2. Access Size and Site

Until December 2021, we performed both standard (30 Fr) and mini (22 Fr) PCNL approaches in ECIRS cases. The approach selection has mainly been based on the stone burden and location, as well as the surgeon’s preference. Since January 2022, we have performed all ECIRS cases through a 22 Fr access sheath (mini PCNL). As we use an 18 Fr rigid nephoscope, the Lithoclast Trilogy^®^ (EMS Medical, Nyon, Switzerland) lithotripter with a smaller probe can still be inserted through it during mini PCNL. In their systematic review, Thapa et al. reported that mini PCNL was associated with a slightly longer operative time but with fewer complications and similar outcomes to the standard PCNL [30]. Likewise, in the randomized controlled trial (RCT) by Wishahi et al., mini PCNL was associated with a lower hemoglobin drop, shorter hospitalization time, and less postoperative pain and need for analgesics compared to standard PCNL. On the contrary, standard PCNL was associated with higher complication rates. The authors reported that SFRs and total operative time did not present significant differences, when considering the non-standardization of the cases, regarding stone characteristics [31]. Hamamoto et al. evaluated the feasibility, safety, and efficacy of mini ECIRS and reported that they were associated with better outcomes than both standard and mini PCNL alone [25]. As already mentioned, in our study, the mean total operative time was similar between standard and mini PCNL (46.55 ± 16.96 min vs. 53.80 ± 17.85 min, *p* = 0.125). The mean hemoglobin drop, SFRs, and complication rates were comparable between the two groups. Nevertheless, it is difficult to isolate the effect of instruments’ miniaturization on outcomes during ECIRS due to the heterogeneity of the stone characteristics and the multiplicity of different equipment and instruments used. Thus, our small sample size and the abovementioned confounding factors limit the strength of our findings. Larger-scale and well-designed studies, such as RCTs, are needed to draw safer conclusions. It is our belief though that the miniaturization of instruments during ECIRS is associated with patients’ faster recovery and less postoperative pain.

Regarding the access site, all cases were performed with a nonpapillary puncture. We have previously reported in an RCT that our nonpapillary technique is not associated with higher blood loss or transfusion rates when compared with the respective PCNL approach to the fornix of the papilla [32]. The nonpapillary approach provides us with an increased range of motion of the nephroscope, which allows more effortless instrument maneuvers to efficiently assess the pelvicalyceal system [32]. Furthermore, the number of punctures that are required to achieve optimal SFRs is reduced when compared to the conventional papillary approach [32]. All punctures were performed near the middle or the lower calyces.

### 4.3. Laser Settings and Type

When the nephroscope was unable to reach the stones, retrograde lithotripsy with a laser device was carried out. As already mentioned, energy settings ranged from 1 to 2 J, while the frequency ranged from 30 to 60 Hz. We routinely perform high-power laser lithotripsy for almost all urolithiasis cases in our center. Our high-power technique, also called the “self-popping” technique, has been described in a previous publication by our group. We have shown that our high-power “self-popping” technique is both safe and efficient and associated with significantly shorter operative times and similar SFRs compared to the conventional low-power “dusting technique” [19]. However, optimal laser settings during lithotripsy represent a controversial topic today. In their comparative study of high- versus low-power laser lithotripsy in lower pole stones during URS, Pietropaolo et al. reported that high-power settings could reduce both the operative time and the need for UAS and postoperative stenting [33]. They also demonstrated that high-power settings were associated with non-statistically significant lower SFRs and reduced sepsis-related complications. Likewise, Chen et al. compared high- and low-power Holmium-YAG settings during multitract mini PCNL for large staghorn stones in an RCT and reported that high-power settings significantly reduced the operative time without increasing complication rates [34]. Regarding the energy source during the PCNL time of the ECIRS procedures, we used the Lithoclast Trilogy^®^ (EMS Medical, Nyon, Switzerland) lithotripter in all cases. By using dual-energy lithotripters with integrated suction, optimal SFRs and reduced total operative time were achieved. The same device with a small probe can be inserted in the 18 Fr nephroscope during mini PCNL [35]. As already mentioned, retrograde laser lithotripsy was performed with either a Holmium-YAG laser or a TFL. The mean operative time was significantly shorter with Holmium-YAG (48.33 ± 16.11 min) than with TFL (62.15 ± 19.99 min), *p* = 0.011, while hemoglobin drop, SFRs, and complication rates were comparable between the two groups. The strength of our findings is, however, limited by the small sample size and the effect of various confounding factors. Moreover, as presented in Table 2, the mean stone size did not differ significantly between the two groups. Nevertheless, in a systematic review and meta-analysis comparing TFL and Holmium-YAG laser lithotripsy, Chua et al. reported that TFL was associated with shorter operative and laser utilization times, while SFRs, complication rates, and length of hospitalization were comparable between the two laser types [36].

### 4.4. ECIRS Indications

As recommended by the EAU guidelines on urolithiasis, most patients with large renal stones still undergo PCNL [37]. In our center, ECIRS is performed in the following cases: (1) stones in calyces that are not reachable with the nephroscope. Performing ECIRS in these cases enables complete laser fragmentation via a retrograde manner or the reposition of these stones in a more suitable location that can be reached and extracted with the nephroscope via the antegrade access sheath. This latest maneuver is called “pass the ball” and has been shown to lessen the number of percutaneous punctures and the probability of iatrogenic ureteric trauma [38]. (2) stones in patients with difficult pelvicalyceal system angles and renal anatomy anomalies. It is worth mentioning that during our five-year experience, we have performed ECIRS in five patients with horseshoe kidneys, three patients with malrotated kidneys, and five patients with duplicated pelvicalyceal systems. In these complex cases, the use of the energy device via the antegrade access and the “pass the ball” maneuver significantly facilitated the procedures, reducing operative time and optimizing SFRs. (3) stones in locations where additional access represents a great risk. The best example of this indication is a stone that is in the upper calyces over the 11th rib [39]. In these cases, minimizing the number of percutaneous punctures and reducing large nephroscope maneuvers, especially those with excessive torque, result in a lower hemoglobin drop [14,25]. Our findings of minimal blood loss during ECIRS confirm these findings, which have also been reported by other groups.

### 4.5. Study Limitations

This study shows certain limitations. The small sample size (*n* = 62) could be considered as a limitation. Although we started performing ECIRS five years ago, the rate of cases treated with ECIRS has gradually increased. We now perform two to three ECIRS procedures per month. Another important limitation is that a comparison group was not used, and thus, the comparative analysis of ECIRS versus other urolithiasis treatment modalities, such as PCNL alone, was not performed. Furthermore, both surgeons (E.L. and P.K.) are highly experienced stone surgeons, and our department represents a high-volume stone center. It is thus possible that optimal outcomes reported in this study are associated with the expertise of the surgeons. Moreover, the comparative analyses between the Holmium-YAG and TFL laser devices and between the standard and mini PCNL techniques are limited by several potential confounding factors, while a propensity-score-matched analysis was not performed and was not the purpose of the current study. Finally, SFRs were mostly defined by KUB X-ray and US, since NCCT was only performed in 32.8% (*n* = 21) of the patients. Following that rationale, some stone fragments could potentially have been missed. Nevertheless, all symptomatic patients and patients with abnormal findings on KUB X-ray or US underwent NCCT.

## 5. Conclusions

Nonpapillary prone ECIRS is a feasible, safe, and efficient approach in patients with specific stone and anatomy characteristics. In our cohort, the total SFR was calculated to be 90.3%, while only nine patients presented with postoperative, transient, Grade II complications. Technical modifications were proposed, and further strength was added to our previous findings [17] with the enrollment of 29 additional patients. The holmium-YAG laser type was associated with shorter operative times. The implementation of more, higher-evidence studies, such as comparative prospective ones and RCTs, is of utmost importance so that safer conclusions can be drawn.

## Figures and Tables

**Table 1:** Patients’ baseline, intraoperative and postoperative characteristics. SD: standard deviation; ASA: American Society of Anesthesiologists classification; BMI: body mass index; TFL: thulium fiber laser; IQR: interquartile range; Hb: hemoglobin; SFR: stone-free rates.

Total Cases, *n* = 62	
Patients’ Baseline Characteristics	
Age, years (mean ± SD)	54.44 ± 12.39
Gender, *n* (%)	Male, *n* = 28 (45.2%)
	Female, *n* = 34 (54.8%)
ASA score, *n* (%)	0, *n* = 3 (4.8%)
	1, *n* = 15 (24.2%)
	2, *n* = 15 (24.2%)
	3, *n* = 29 (46.8%)
BMI, kg/m^2^ (mean ± SD)	26.83 ± 3.51
Stone size (maximal diameter), mm (mean ± SD)	39.03 ± 13.93
Staghorn, *n* (%)	*n* = 15 (24.2%)
Anatomical abnormalities, *n* (%)	Horseshoe kidney, *n* = 5 (8.1%)
	Malrotation, *n* = 3 (4.8%)
	Duplicated system, *n* = 5 (8.1%)
Intraoperative Characteristics	
Operative time, minutes (mean ± SD)	51.23 ± 17.75
Access number, *n* (%)	1, *n* = 48 (77.4%)
	2, *n* = 10 (16.1%)
	3, *n* = 4 (6.5%)
Access size, *n* (%)	22 Fr, *n* = 40 (64.5%)
	30 Fr, *n* = 22 (35.5%)
Access site, *n* (%)	Middle calyces, *n* = 13 (21.0%)
	Lower calyces, *n* = 49 (79.0%)
Laser type, *n* (%)	Holmium-YAG, *n* = 49 (79.0%)
	TFL, *n* = 13 (21.0%)
Postoperative Characteristics	
Hospital stay *, days (median, IQR)	3.0 (2.0–4.0)
Hb drop, g/dL (mean ± SD)	1.23 ± 0.54
SFR, *n* (%)	*n* = 56 (90.3%)
Complications, *n* (%)	*n* = 9 (14.5%)
Additional treatment, *n* (%)	*n* = 5 (8.1%)

* Hospital stay was expressed as median because its distribution was non-symmetrical (skewed).

**Table 2:** Association of laser type with outcomes. SD: standard deviation; TFL: thulium fiber laser; Hb: hemoglobin; SFR: stone-free rates.

Laser Type	Holmium-YAG	TFL	*p*-Value
Operative time, minutes (mean ± SD)	48.33 (±16.11)	62.15 (±19.99)	*p* = 0.011
Stone size (maximal diameter), mm (mean ± SD)	37.35 (±12.37)	45.38 (±17.85)	*p* = 0.064
Hb drop, g/dL (mean ± SD)	1.28 (±0.48)	1.03 (±0.73)	*p* = 0.144
SFR, *n* (%)	*n* = 44 (89.80%)	*n* = 12 (92.31%)	*p* = 0.785
Complications, *n* (%)	*n* = 7 (14.29%)	*n* = 2 (15.38%)	*p* = 0.920

**Table 3:** Association of access size with outcomes. SD: standard deviation; Hb: hemoglobin; SFR: stone-free rates.

Access Size	30 Fr (Standard PCNL)	22 Fr (Mini PCNL)	*p*-Value
Operative time, minutes (mean ± SD)	46.55 (±16.96)	53.80 (±17.85)	*p* = 0.125
Stone size (maximal diameter), mm (mean ± SD)	38.58 (±14.13)	39.86 (±13.85)	*p* = 0.731
Hb drop, g/dL (mean ± SD)	1.17 (±0.23)	1.26 (±0.66)	*p* = 0.548
SFR, *n* (%)	*n* = 20 (90.91%)	*n* = 36 (90.0%)	*p* = 0.908
Complications, *n* (%)	*n* = 3 (13.64%)	*n* = 6 (15.0%)	*p* = 0.884

## Data Availability

All data generated during this study were analyzed and the results were included in this article. The data presented in this study are available on reasonable request from the corresponding author.
